# Supplementary figures and images for: Response of the subalpine bunchgrasses to wildfires and its effects in the relative abundance of the volcano rabbit in the Ajusco-Chichinautzin Mountain Range
Source: PeerJ. 2024 Jun 28;12:e17510. doi: 10.7717/peerj.17510 (PMC11216220; doi:10.7717/peerj.17510)

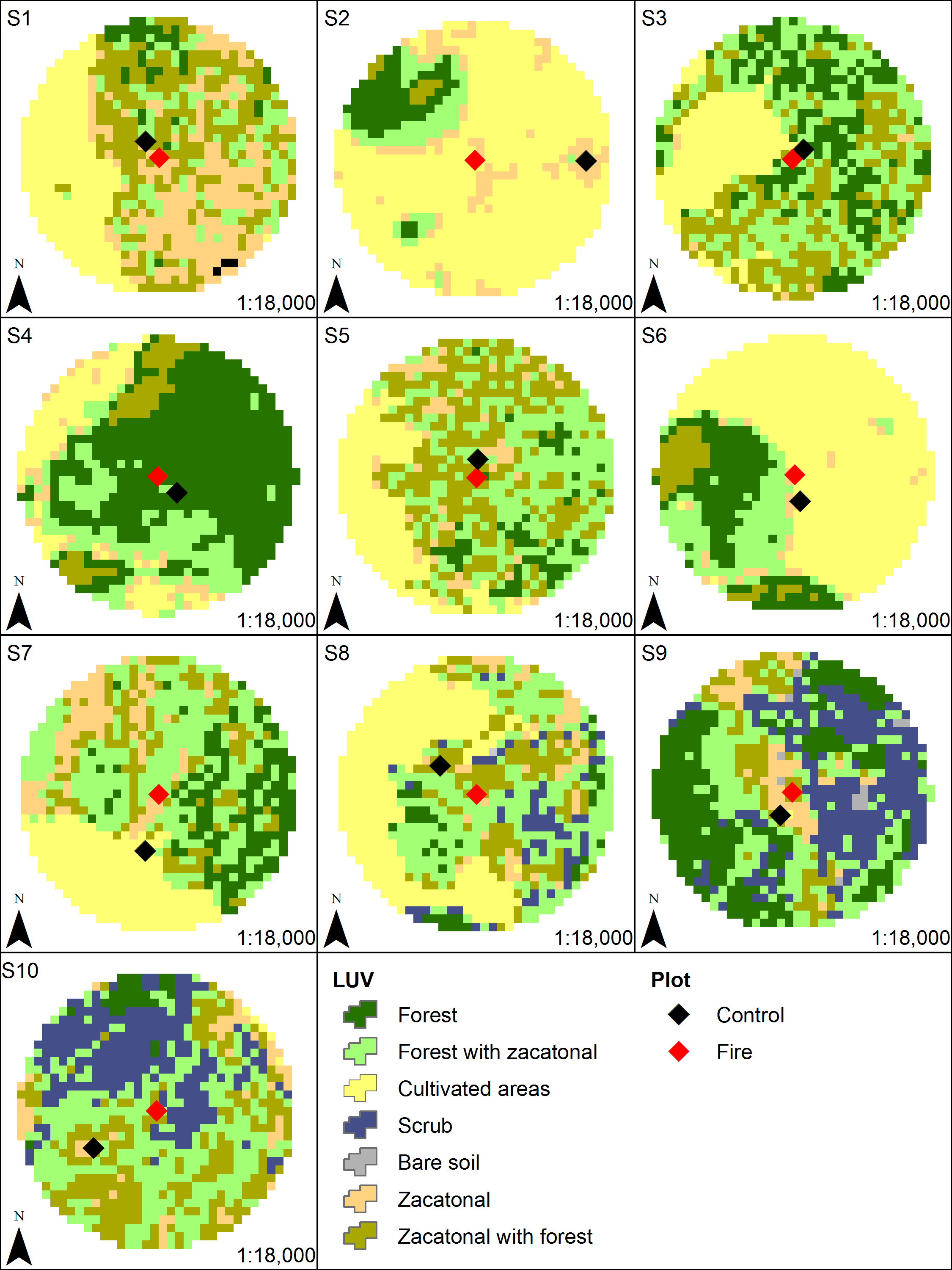

Supplement: Supplemental Information 1 — Burnt plots are represented in red and unburnt plots are in black. [file peerj-12-17510-s001.png]

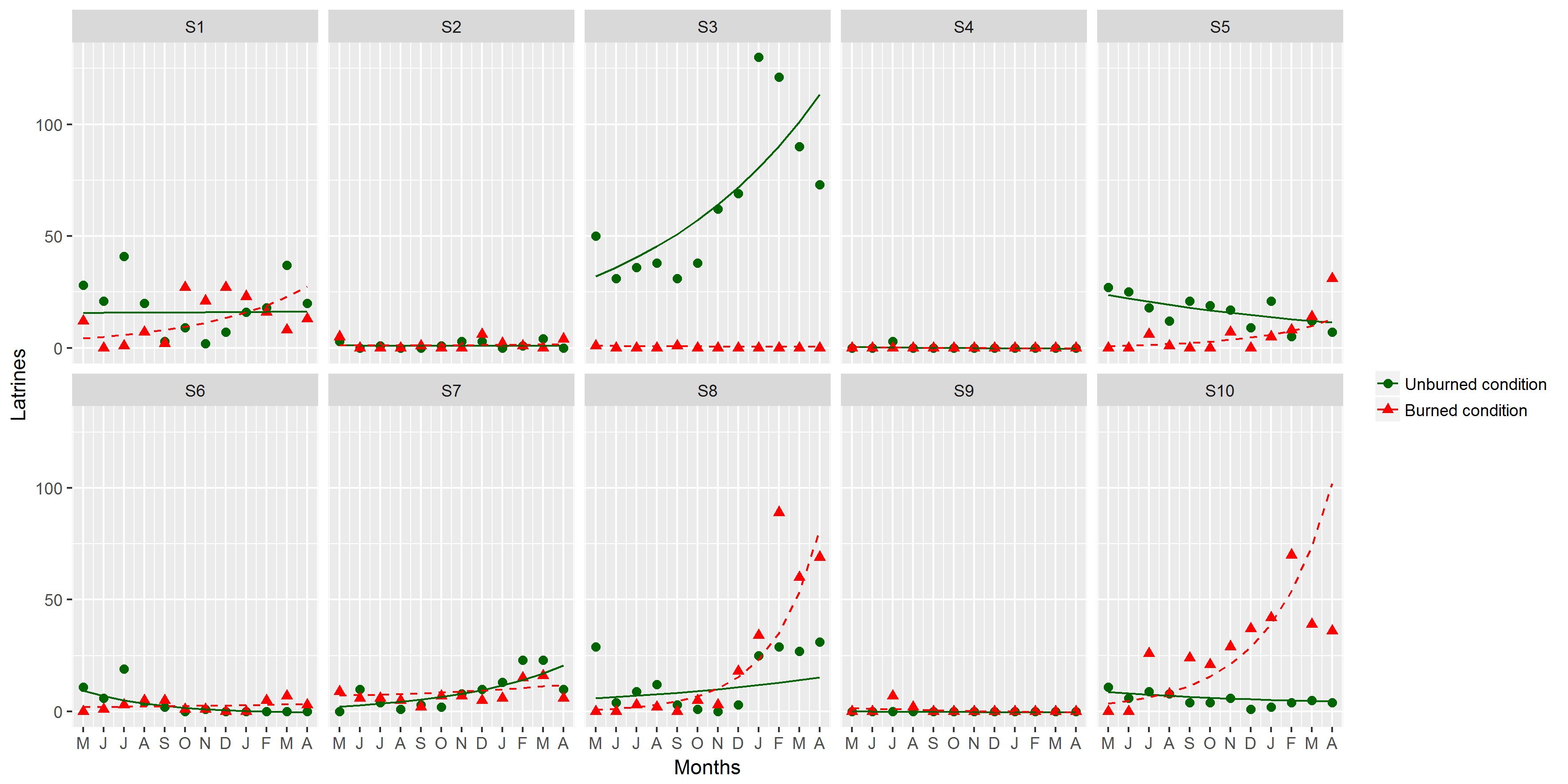

Supplement: Supplemental Information 2 — The lines were estimated using the coefficients generated by the Linear Mixed-Effects Model. Unburnt plots are shown in green and burnt are in red. Months are in chronological order, starting in May 2016 and culminating in April 2017. [file peerj-12-17510-s002.png]

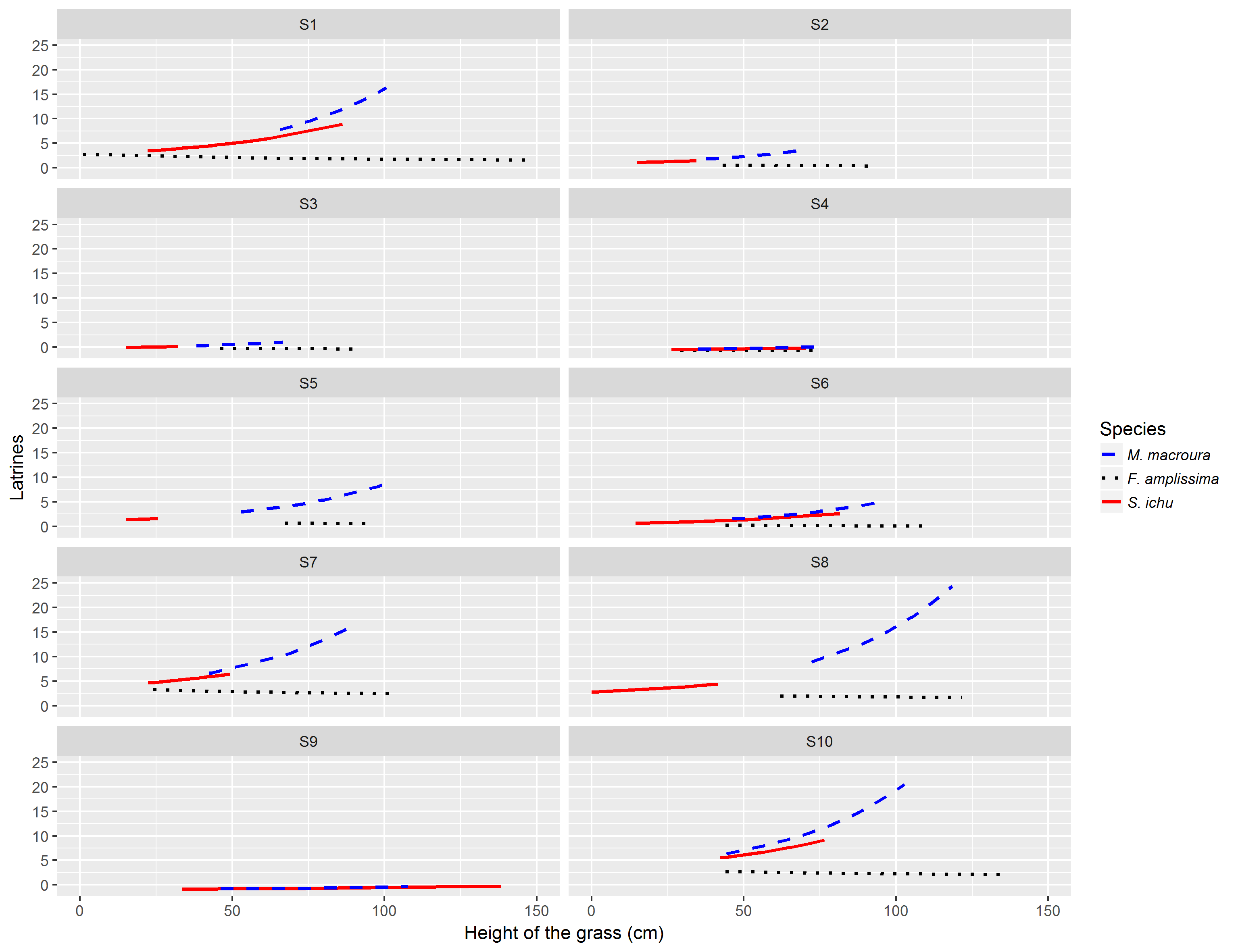

Supplement: Supplemental Information 3 — The graphs show the estimated values of the number of latrines in response to the height of the bunchgrasses based on the coefficients obtained from the Generalized Linear Model. [file peerj-12-17510-s003.png]
