# Supplementary material for: Response of the subalpine bunchgrasses to wildfires and its effects in the relative abundance of the volcano rabbit in the Ajusco-Chichinautzin Mountain Range
Source: PeerJ. 2024 Jun 28;12:e17510. doi: 10.7717/peerj.17510 (PMC11216220; doi:10.7717/peerj.17510)
Supplement: Supplemental Information 7 — The total number of latrines over the twelve months is presented per plot. The third column contains the total number of latrines per site (both plots). [file peerj-12-17510-s007.docx]

|  | Number of latrines in the  unburnt plot | Number of latrines in the  burnt plot | Total number of latrines  per site |
| --- | --- | --- | --- |
| Site 1 | 222 | 157 | 379 |
| Site 2 | 16 | 19 | 35 |
| Site 3 | 769 | 2 | 771 |
| Site 4 | 3 | 0 | 3 |
| Site 5 | 193 | 72 | 265 |
| Site 6 | 43 | 31 | 74 |
| Site 7 | 107 | 90 | 197 |
| Site 8 | 173 | 283 | 456 |
| Site 9 | 0 | 9 | 9 |
| Site 10 | 64 | 332 | 396 |
| Mean | 159 | 99.5 | 258.5 |
