# Supplementary material for: Response of the subalpine bunchgrasses to wildfires and its effects in the relative abundance of the volcano rabbit in the Ajusco-Chichinautzin Mountain Range
Source: PeerJ. 2024 Jun 28;12:e17510. doi: 10.7717/peerj.17510 (PMC11216220; doi:10.7717/peerj.17510)
Supplement: Supplemental Information 8 — Showing the percentage of variance explained by each principal component (PC) and the loadings of the variables associated with the burnt plots in the 10 sites evaluated. The highest correlation positive and negative values are in bold. [file peerj-12-17510-s008.docx]

|  | PC1 | PC2 | PC3 | PC4 | PC5 | PC6 |
| --- | --- | --- | --- | --- | --- | --- |
| *Variance explained* | *0.3537* | *0.1908* | *0.1844* | *0.1308* | *0.0646* | *0.0407* |
| Fire | 0.1685 | -0.2561 | **-0.4674** | -0.0206 | **0.6446** | **-0.5002** |
| Bunchgrass | -0.0480 | -0.1191 | **0.5809** | **0.4372** | -0.0541 | -0.4614 |
| Bunchgrass with forest | 0.3737 | **-0.4339** | 0.1823 | 0.0805 | -0.021 | -0.0235 |
| Forest with bunchgrass | 0.4262 | -0.1623 | -0.123 | -0.3834 | -0.2705 | 0.1015 |
| Forest | -0.0038 | 0.4236 | -0.4638 | 0.2998 | -0.3837 | -0.3426 |
| Cultivated area | **-0.4749** | -0.1933 | 0.0017 | -0.0008 | 0.3187 | **0.3530** |
| Bare soil | 0.1920 | **0.5441** | 0.2131 | 0.1758 | **0.4760** | 0.1067 |
| Scrub | 0.2550 | 0.4335 | 0.2220 | -0.4327 | 0.1679 | -0.0271 |
| Diversity | **0.4822** | -0.0685 | 0.1421 | 0.1347 | 0.0178 | 0.0549 |
| Distance between plots | -0.2969 | 0.0114 | 0.2563 | **-0.5734** | -0.0641 | -0.5186 |
